# On Lake Erie as a Water Supply for Towns on Its Borders1Read before the Microscopical Club of the Buffalo Society of Natural Sciences, January 13, 1896.

**Published:** 1896-08

**Authors:** George W. Rafter

**Affiliations:** Rochester, N. Y.


### ON LAKE ERIE AS A WATER SUPPLY FOR THE TOWNS ON ITS BORDERS.[1]

By GEORGE W. RAFTER, M., Am. Soc., C. E., Rochester, N. Y.

INVESTIGATE the matter in whatever direction we may and we reach always the same result, that in the art of making life safe as well as enjoyable the European cities are far in advance of our own. The time was when in the water supplies, at any rate, the foreign cities were not much better than ours; the water supplies on the other side were like our own, mostly bad, but in the last ten or fifteen years a great change has taken place, until now one may travel to about every large European city drinking the water as furnished at every stop without any anxiety as to the effect on health.[2] With us, on the contrary, a really safe water supply is the exception—leaving out of the account a few cities—and we may say there are as yet almost no sanitarily unobjectionable water supplies in the United States.

As to just how discreditable this is to our cities I do not now attempt to determine. I merely point out the indisputable fact, leaving to others the task of determining the final causes. Before proceeding to the main subject of this paper—namely, Lake Erie as a water supply for the towns on its borders, I wish to give a brief account of two European water supplies, which among others I had an opportunity to study somewhat while abroad last year.

The first of these is that of the city of Liverpool. In 1847 Liverpool was referred to in a government sanitary report "as the most unhealthy town in the United Kingdom." The death-rate then was 63.5 per 1,000. At present it is 26.1 per 1,000. Previous to 1847 the water supply was in the hands of two private companies whose franchises dated from 1799 and 1822. Both of these companies distributed water to their consumers on the intermittent system twice or three times a week and for not more than two or three hours at a time. In this system each house is fitted with a tank in the attic, of a capacity graduated to the number of people living in the house. The pressure is only on the street mains at certain definite seasons, at which time each householder must see that his supply-pipe valve is opened, as otherwise the house must remain without water until the next stated period for filling the tanks comes around.

---

1. Read before the Microscopical Club of the Buffalo Society of Natural Sciences, January 13, 1896.

2. Allen Hazen's Filtration of Water Supplies. The author's observations are to the same effect on this point.

In 1847, however, these private companies conveyed their franchise to the corporation of Liverpool, and in the same year the corporation was authorised by act of parliament to carry out what is known as the Rivington Pike Water Supply designed by the great English engineer, Thomas Hawksley. This was a project for impounding the headwaters of the rivers Douglas, Yarrow and Roddlesworth by the construction of large reservoirs in a hilly and sparsely settled district about twenty-five to thirty miles away. These works were finished in 1857.

The Rivington Pike Works include six impounding reservoirs with a total capacity of 4,200,000,000 imperial gallons and a catchment area of 10,000 acres. The one fact to which I wish to call special attention is this, that although the Rivington Pike Supply is gathered from a sparsely settled, hilly country, ranging in elevation above the sea level from 425 to 1,500 feet, still this water, from an area naturally superior even to the Hemlock lake area of the Rochester water supply, is all filtered. The original design included the preparation of 6.25 acres of filter areas as a necessary part of the project. Ever since 1852, when these works were first started, or for forty-three years, it has been recognised at Liverpool that any water, subject even remotely to sewage contamination, is unsafe without efficient sand filtration. In 1873 it was felt that the Liverpool water supply should be extended, and after much discussion it was decided to construct an artificial lake in the valley of the River Vyrnwy, in Wales, and to bring the water from thence by iron mains to Liverpool, a distance of sixty-eight miles. This great work was finally completed and opened for use in 1892.

Lake Vyrnwy is situated in a remote and unfrequented part of Wales, with neither a railway station nor town of any size within ten miles. The nearest railway station is Llanfyllin, which may be reached via Oswestry, from either Birmingham or Shrewsbury to the east, or from Liverpool and Chester from the north. A drive from this place to Lake Vyrnwy is an experience worth going abroad to take, especially if, as in my case, a typical Welsh rainy day happens to be encountered. The Vyrnwy dam has a height of 161 feet from foundation to parapet of the carriage road crossing it. The height from river bed to sill of overflow weir is eighty-four feet. The lake created by this immense structure is 4.75 miles in length and an average of one-half mile in width. The area of the water surface when full is 1,121 acres, and the storage 2,103,000,000 cubic feet, equivalent to 13,125,000,000 imperial gallons.

The catchment area is 18,000 acres, but the Liverpool corporation have power to divert two neighboring streams with catchment areas of 5,200 acres, so that when all the works contemplated by the act of parliament have been carried out, the lake will have a total watershed of 23,200 acrès. The total cost of the Vyrnwy works to 1882 was roundly £2,100,000. Please bear in mind that this expenditure was for an extension of the works merely. It does not include any part of the cost of the Rivington Pike or original water companies' supply. As stated, the catchment area of Lake Vyrnwy proper comprises about 18,000 acres, of which the Liverpool corporation owns about 12,500 acres. It is intended to ultimately purchase the balance of the area which is now included in two estates.

The catchment area, generally, is very sparsely populated, and on that portion owned by the corporation, the sanitary arrangements of residences, barns and barnyards have been remodeled wherever necessary to protect the issuing water. Nevertheless—and here again is the point of the whole matter—the Vyrnwy supply is all filtered, the same as the Rivington Pike supply before it goes to the consumer.

At Oswestry, about twenty miles from the lake, at a point on the conduit line, where a convenient level area exists, the water is taken out and subjected to efficient sand filtration.

At the present time the possible daily supply is 32,000,000 imperial gallons, but the Vyrnwy works are so arranged with reference to future extension as to admit of a daily supply, ultimately, of 58,500,000 gallons.

The actual consumption for a population of 870,000, is 20,-000,000 gallons per day, giving a per capita consumption of twenty-three gallons. This we may bear in mind is imperial gallons, equivalent to 27.6 U. S. gallons per day.

In this connection it may be remarked that the population of the city of Liverpool itself is only about 500,000. The corporation is, however, required by statutory provisions to supply certain adjacent districts, thus bringing the total of the population supplied up to about 870,000, as stated.

The following are some analyses of the Liverpool water as it goes to the consumer :

TABLE NO. 1.—CHEMICAL ANALYSES OF THE LIVERPOOL WATER SUPPLY.

(In parts per 100,000.)

| | Total solid impurity. | Organic carbon. | Organic nitrogen. | Ammonia. | Nitrogen as nitrites & nitrates. | Total combined nitrogen. | Chlorine. | Total hardness. | |
|---|---|---|---|---|---|---|---|---|---|
| VYRNWY SUPPLY. | | | | | | | | | |
| Av'ge winter water after filtration .. | 4.68 | 0.361 | 0.024 | 0.0 | 0.0 | | 0.74 | 2.9 | Clear and yellow. |
| Summer water after filtration ....... | 4.39 | 0.198 | 0.023 | 0.002 | 0.0 | 0.025 | 0.77 | 2.7 | Clear and pale yellow. |
| Rivington Pike.... | 9.66 | 0.210 | 0.029 | 0.002 | 0.0 | 0.031 | 1.53 | 4.0 | Clear. |

Probably, however, the best index as to the purity of a municipal water supply is that derived from the typhoid fever death-rate. For the years 1890 and 1891, the rate for Liverpool was 1.8 deaths per 1,000 of the population. The death-rate from all causes in these years was 27.7 per 1,000 of the population.

In considering the significance of this latter figure we need to understand that Liverpool has the greatest density of population per acre of any city in Great Britain. Thus the density of population in London is 56.5 persons per acre; Birmingham, 57.2; Bristol, 61.8; Manchester, 39.6; Glasgow, 92.8 and Liverpool, 99.3, as determined from the census of 1891.

The second European municipal water supply, to which I wish to briefly call attention, is that of the city of Zurich, Switzerland.

The original water supply of Zurich was taken from springs, but in 1873 the original supply having proven too small, it was resolved to also take a supply from the River Limmat, which is the outlet of Lake Zurich. The river water first passed from the river into a submerged filter area, constructed in the bed of the river; whence the filtered water flowed through a concrete conduit to the pumping station. In the months of March, April and May, 1884, Zurich was visited by a virulent outbreak of typhoid fever, during which time fully 2 per cent. of the population were attacked and 9 per cent. of the cases proved fatal; thus giving the high rate of 180 deaths in 100,000 population.

A commission appointed to inquire into the cause of this epidemic, reported that the infection could not be traced to abnormal meteorological or sanitary conditions, but that the filtered river water, although clear and satisfactory in appearance as well as chemically of a high degree of purity, still contained an abnormal number of bacteria. On investigation it was discovered that the submerged filter bed and the concrete main leading therefrom to the pumping station were both so seriously defective as to allow the raw water of the river to pass to the pumps, and thence to the reservoirs and so finally to the consumers. The concrete conduit had been injured by blasting in the river bed near by to remove an obstructing rock. The defective filter and conduit had both passed unnoticed, because the river was normally so clear and chemically pure that not only the officers in charge of the water supply, but the citizens generally, believed that the filtration was really a matter of little importance. It was also ascertained that about the time of the outbreak extensive dredging operations, in connection with the new quay works at the lake and along the river, had been carried on whereby the bottom had been thoroughly stirred up.

These two facts in conjunction with the results of the bacteriological investigations strongly indicated the river water as the vehicle of infection; the more so, because at Geneva, where similar dredging operations were in progress, there was at the same time a severe epidemic of typhoid fever. The result of this experience was that Zurich immediately recognised the necessity for a new water supply. In the meantime the area to be dredged was entirely enclosed with a coffer-dam and a cast iron conduit with lead joints substituted for the defective concrete main of the old supply. The new supply was taken from the lake itself rather than from any of the springs or streams of the adjacent alpine region. The result of the typhoid epidemic had so far awakened the people of Zurich that in any case it was deemed essential to the purity of any new supply that it should be filtered.

A bacteriological investigation showed that while the mud at the bottom of the Swiss lakes frequently swarmed with bacteria, still the water at mid-depth was usually very pure. Some of the results obtained from cultures are as follows: In the Lake of Zurich, at the foot, 170 per cubic centimeter; in Lake Luzerne, 50; in Geneva, 38; and in Constance, 58 per cubic centimeter. The spring water of the vicinity after a rain frequently contains as high as 2,000 bacteria per cubic centimeter.

Lake Zurich is about twenty-five miles in length and nearly two miles wide. The catchment area is 423 square miles. Its mean depth is 230 feet, and the maximum 475 feet. The mean discharge of the River Limmat is about 3350 cubic feet per second.

The Zurich authorities consider that the self-purifying capacity of the Lake of Zurich is considerable and formulate about the following, as expressing the various causes the self-purification is due to:

(1) First, sedimentation, which is favored by the slow motion of the water.

(2) By its freedom from severe agitation, which prevents matter once at the bottom from being stirred up every few days. The lake is surrounded on all sides by high mountains which prevent severe wind action. At the same time there is wind action enough to assist oxidation at the surface without such severe agitation as stirs up the bottom.

(3) By the great depth which further tends to keep organic matter at the bottom after it once reaches there.

(4) The Swiss lakes all possess an aquatic founa of great variety, which, collecting at the mouths of drains and streams, feeds upon the impure matter brought down.

(5) By reason of the undisturbed condition at the bottom, innumerable quantities of microbes develop, which feed upon the sediment as it reaches them.

The difficulty is to know where the self-purifying process is complete ; hence, as an additional safeguard, the Zurich authorities decided to also filter the water by the most efficient methods of sand filtration. The new works were therefore designed with a filter station as an integral part of the plant.

I will not consume space in describing the mechanical arrangements of the new works with filter areas, because that is more appropriately discussed as a matter of engineering construction. At present we are chiefly concerned with the physical, chemical and biological considerations which should guide us in deciding whether a given body of water is a fit source for a municipal supply. The population of Zurich is, with its suburbs, about 100,000, and the water supply is placed at about eighty U. S. gallons per capita per day. The investigations carried on in the municipal laboratory and at the Hygienic Institute of Zurich have developed a number of important principles, to some of which we may refer a little in detail.

As to the effect of the freezing over of the lake on the quality of the water.

In 1880 the winter was exceptionally severe and when the ice on the lake broke up in the spring, there was a considerable increase of typhoid cases, which, after the epidemic and investigation of 1884, was attributed to a probable contamination of the river water, passing through the old submerged filter and concrete mains. In the winter of 1889-90 the frost was still more severe than in 1880, and it was resolved to make such a study as might throw some light on the quality of the water in winter. The entire lake was frozen over from January 17th to March 20th, during which time a considerable quantity of impure matter had accumulated on the surface of the ice, at and about the foot of the lake, which it was feared might contaminate the water supply when the ice broke up. A series of special analyses were therefore made between January and May with the view of ascertaining whether, and how far, the quality of both the unfiltered and filtered water would be affected during the frost, and during and after the thaw. Table No. 2, compiled by Mr. Preller for his paper on the Zurich water works,[1] gives the result of these analyses.

TABLE NO. 2.—SHOWING THE EFFECT OF FREEZING OVER OF THE LAKE ON THE QUALITY OF THE WATER AT ZURICH.

| Chemical Constituents expressed in parts per 100,000. | November 15, 1889, to January 17, 1890. Normal. | January 18, 1890, to March 19th. Lake frozen over. | March 20th to April 5th. Lake open. | April 6th to May 12th. Intermediate period. | May 13th to June 27th. Normal. |
|---|---|---|---|---|---|
| BEFORE FILTRATION: | | | | | |
| Organic matter | 1.69 | 1.83 | 1.77 | 1.80 | 1.83 |
| Free ammonia | 0.0007 | 0.0009 | 0.0007 | 0.0005 | 0.0005 |
| Albuminoid ammonia | 0.0028 | 0.0032 | 0.0030 | 0.0029 | 0.0033 |
| BACTERIA (per cubic centimeter): | | | | | |
| Mean number for period | 122 | 667 | 938 | 632 | 171 |
| Maximum number for period | 202 | 2179 | 2152 | 1425 | 229 |
| TEMPERATURE (F°): | | | | | |
| Mean of water | 41°.4 | 37°.4 | 39°.0 | 40°.0 | 48°.2 |
| Mean of air | 15°.0 | 16°.0 | 40°.0 | 45°.0 | 57°.2 |
| AFTER FILTRATION: | | | | | |
| Organic matter | 1.41 | 1.44 | 1.40 | 1.41 | 1.49 |
| Free ammonia | 0.0 | 0.0 | 0.0 | 0.0 | 0.0 |
| Albuminoid ammonia | 0.0018 | 0.0019 | 0.0019 | 0.0020 | 0.0019 |
| BACTERIA (per cubic centimeter): | | | | | |
| Mean number for period | 10 | 13 | 9 | 11 | 16 |
| Maximum number for period | 20 | 27 | 29 | 22 | 21 |

3 Proc. Inst. C. E., Vol. CXI., p. 281.

We may draw the following conclusions from Table No. 2 :

(1) The freezing over of the lake was followed by a large increase in the number of bacteria which continued until the ice broke up.

(2) The breaking of the ice was followed by a still greater increase, temporarily, due to the passage into the water of the large amount of filth which had collected on the surface.

(3) After the break-up the water gradually returned to substantially the same condition as at the beginning of winter.

(4) The fluctuation in number of bacteria are apparently independent of temperature, although not necessarily so, as the decrease in temperature may have reached the point just as the lake closed where the annual overturning occurs by which the bottom water is brought to the top. As we shall see further on, the number of bacteria is greater at the bottom than elsewhere.

(5) We may say, however, that the great increase in bacteria during the frost period is due probably to the impeding of the natural process of oxidation by the exclusion of air from the water.

On this point we may note that the aërobian forms are antagonistic to the anaërobians, whence the anaërobians may be expected to multiply more rapidly with the lake frozen than when open. We find here a rational reason why typhoid fever would naturally become epidemic with the lake frozen.

(6) We note, too, from Table No. 2, that throughout these bacteriological fluctuations, the water shows but slight and inappreciable chemical changes from its normal condition. We find them a strong reason why the bacteriological investigation is of supreme importance.

(7) We also learned that the filtered water hardly varied in quality during the whole period.

As to the proper depth of the intake in such a lake.

On this point, chemical and bacteriological examinations were made at various depths, as shown by Table No. 3.

TABLE NO. 3.—PRINCIPAL CHEMICAL CONSTITUENTS IN PARTS PER 100,000 AND NUMBER OF BACTERIA IN UNFILTERED LAKE WATER AT VARYING DEPTHS.

| | 13 Feet. | 39 Feet. | 46 Feet. | 52 Feet. |
|---|---|---|---|---|
| Organic matter | 2.11 | 2.16 | 2.16 | 2.17 |
| Free ammonia | 0.0006 | 0.0011 | 0.0011 | 0.0011 |
| Albuminoid ammonia | 0.0046 | 0.0044 | 0.0044 | 0.0048 |
| Bacteria (per cubic centimeter) | 149 | 160 | 175 | 352 |

The intake is actually set at the depth of forty-two feet, while the total depth at the point of intake is fifty-six feet. Table No. 3 shows that the quality of the water is not impaired by greater depth of intake up to forty-six feet; but at fifty-two feet the increase in number of bacteria points to the influence of the large number living at and near the bottom.[1]

In considering Lake Erie as a source of water supply for the towns on its borders, we shall see that the results of the experience studies abroad may be beneficially applied here.

The length of Lake Erie is about 240 miles and its mean breadth a little less than forty miles. The area of the water surfaces is given at 9,522 square miles, while the catchment area is 21,370 square miles.

The average depth in the western portion is about thirty feet; off Cleveland the average depth is about sixty to seventy feet with a maximum of about ninety feet. The deepest portion is well out in the middle between Erie and Dunkirk, where a maximum depth of 240 feet is attained, but the area of this depth is very small. The average depth of the whole lake may be taken at sixty feet. We have here a shallow lake with, generally speaking, low flat shores on every side and hence subject to the full sweep of nearly every prevailing wind of the region. By way of emphasising this last statement it may be observed that it is quite common experience in severe, long-continued storms, when the wind sets from the west end of the lake toward the east, that the waters pile up several feet, doing considerable damage to the lower part of the city of Buffalo. In winter, on account of its shallowness, Lake Erie is more or less closed with ice, sometimes months at a time.

Referring to the hydrographic charts of Lake Erie and we find that for nearly the whole area the bottom is described as clay, although there are occasional small areas of sand and gravel.

I will not take time to give statistics of population in the Lake Erie catchment area and the consequent amount of sewage which daily finds its way into its waters. It is enough to say that the populous cities of Detroit, Toledo and Cleveland, as well as a number of smaller towns, all send their sewage, absolutely without treatment, to mingle with its waters; from which again Cleveland, Erie, Dunkirk, Buffalo and other towns take their water supplies—that is to say, the citizens of these several cities complacently drink raw water containing raw sewage.

---

1. A more extended description of the Zurich Water Works may be found in Mr. Preller's paper in the Proceedings of the Institution of Civil Engineers, already referred to.

The physical characteristics of Lake Erie, as exhibited in the foregoing, are the opposite of those of the Lake of Zurich—namely, the Lake of Zurich is in the heart of the Alps and protected from severe winds by the high mountains on all sides; the opposite applies to Lake Erie. Again, the depth of the Lake of Zurich is such that the slight wave-action produced by the moderate winds of the region hardly ever penetrates to the bottom; while in shallow Lake Erie a large portion of the bottom is stirred up with every severe gale.

This frequent stirring up of the bottom of Lake Erie would be far more serious in its effect on the quality of the water supplies if it were not for one modifying condition: the clay silt of the bottom is a good precipitant, and the clarifying process takes place much quicker after the gale is over than it would if the clay were absent.

Still, in any case, this frequent stirring-up of the bottom is serious enough in its effect on the water supplies, because while the gale continues, and for a short time thereafter, the deposited sewage of the previous longer or shorter period of calm is thoroughly commingled with the whole body of water.

There is another phase of this stirring-up process which we may briefly refer to. Dr. Drown has shown that the amount of oxygen in solution in the water of natural ponds and lakes decreases as we go towards the bottom, until, when the bottom is finally reached, it is, in many cases, nearly *nil.* It follows in such cases that the aërobian forms of microbic life must inevitably succumb to the anaërobian forms, which latter, including the bacillus of typhoid fever, have a free field in which to develop to the utmost limit of luxuriance.

If, then, we give the bottom a thorough stirring up occasionally, we certainly bring the reducing action of oxygen and aërobic life to bear upon objectionable organic matter which otherwise would never experience such influence except, in slight degree, at the annual overturning which takes place by reason of change in temperature.

In April and May, 1895, I examined Lake Erie somewhat carefully as a source of water supply, with reference to an extension of the water works of the manufacturing town of Lorain, on the south shore, about twenty miles west of Cleveland. As some of the results of that study are of general interest I will refer to them here.

Lorain is situated at the mouth of the Black river, which is estimated to have a catchment area of 410 square miles. The Black river flows through Elyria, a town of several thousand inhabitants and ten to twelve miles away. This town constructed water works in 1879 and although still without a comprehensive system of sewerage has, nevertheless, sewers in the main streets to which the hotels and principal business blocks have been connected for several years, and which, after the manner of other towns in general, debouch into the stream without treatment, and at what the authorities of Elyria consider the most convenient point, *i. e.*, at the least expensive to them. The net result is that Elyria contributes an appreciable amount of organic filth to the stream.

At Lorain the river is projected into the lake by jetty piers about one-third of a mile in length, while the main outlet sewer of Lorain enters the river near the shore end of the jettys.

The Black river issues from the clay areas of Northern Ohio, and, like all streams from such areas, is subject to extreme variations in its flow. For instance, the extreme dry weather flows are as low as from fifty to 100 cubic feet per second, while flood flows of from 10,000 to 12,000 cubic feet per second are quite common—they may be expected once or twice a year. The extreme flood flows run up to 15,000 to 18,000 cubic feet per second.

There is deep water from the mouth to about five miles back with a cubic content of 70,000,000 cubic feet. Beyond the deep water the river is generally rapid to and beyond Elyria. The sewage of Elyria receives the benefit of the rapid flow of several miles before entering the reach of still water at the foot of the rapids.

The present population of Elyria is about 8,000 and Lorain about 10,000, or the total population now contributing pollution to the deep-water section of the Black river is roundly 18,000, although previous to 1894 the population of Lorain was only about 5,000, but the location there of the Johnson Company's extensive steel plant has resulted in a doubling of the population in a few months.

During low water the velocity in the deep-water reach of the river is so slight that the polluting organic matter quickly settles to the bottom, where we may assume, from our recent advances in knowledge of such matters, that it remains nearly unchanged until the occurrence of a flood flow of sufficient volume to sweep it out into the lake. In this way the accumulation of several months may be swept into the lake in a few hours, where, under certain conditions of winds and currents, it may be disseminated throughout the entire body of lake water for a radius of several miles from the mouth of the river.

In order to illustrate the possible effect of a large flood flow in contaminating a water supply situated like that at Lorain, it is pointed out that a flood flow of 15,000 cubic feet per second amounts to 1,116,000,000 cubic feet in twenty-four hours, a quantity of water sufficient to make ten feet in depth over an area of four square miles, or, what is the same thing, one foot in depth over forty square miles.

The water supply of Lorain is taken from the lake 1,300 feet west of the end of the jetty piers. On inquiring of the water works trustees of that town as to why the crib had been located so near the mouth of the river, I was told they had been advised when the location was made that since the currents in Lake Erie were from the west towards the east, if they went even a short distance west of the mouth of the river they would certainly obtain an unpolluted supply. The foregoing brief statement of some of the physical problems applying to such cases may serve to indicate the fallacy of such conclusion. Indeed, we may say it follows with the certainty of a proposition in mathematics, that a water supply taken in the vicinity of the mouth of a stream like the Black river is liable at times to a serious contamination. On this point extended observations, made in England, as to the relation between floods in the River Tees and the prevalence of typhoid fever in certain towns deriving their water supply from that river, show that in 1890 and 1891, the years in which the observations were made, nearly every flood was followed by a considerable rise in the typhoid fever rate in the towns observed.

The Health Department of the City of Buffalo has, to the credit of that city, instituted frequent systematic bacteriological examinations of the Lake Erie water as supplied to the consumers in Buffalo. By courtesy of Dr. William G. Bissell, city bacteriologist, I have been afforded an opportunity to examine the results of the last year's work. The most interesting point brought out is the great decrease in number of bacteria present in the water as it leaves the reservoir in comparison to the number in the water as it enters. The sedimentation frequently reduces the number from 30,000 to 40,000 per cubic centimeter to from 400 to 700 per cubic centimeter. In a practical way, however, it ought not to be overlooked that the water at the bottom of the reservoir must contain vast numbers, among which it is probable that some are the specific morbific causes of disease. We may conclude, then, that the bottom needs to be cleaned frequently as a safeguard against possible contamination from this source.

The offieials of the Buffalo Health Department are, however, disposed to say that Lake Erie is an unexceptionable source of water supply because they hardly ever detect the specific germ of typhoid fever. On this point I am compelled to disagree and cite the results of an investigation of the relation of the bacillus of typhoid fever to sewage made by Laws and Andrewes for the London County Council, as published in the early part of 1895.[1]

Messrs. Laws and Andrewes state that working on ordinary London sewage they failed in every case to recognise the typhoid bacillus, until finally the failures were so numerous that they became oppressed by a sense of mathematical improbability. Thus the average amount of sewage produced in London amounts to 200,000,000 gallons per day. During June, 1894, while the investigation was in progress, 177 cases of typhoid fever were notified in London, to which may be added thirteen cases of continued fever, making 190 cases in all. Adding something for cases not reported and Messrs. Laws and Andrewes conclude that during June, when typhoid is by no means prevalent, it may be assumed that there were 200 cases in all. Some of these suffer from constipation and hence contribute very little fecal matter to the general mass of sewage. For these and other reasons cited, any estimate of the average amount of sewage contributed daily by typhoid cases in London must be purely conjectural, but at a reasonable estimate it is placed at one two hundred and fifty millionths of the whole, and, as Laws and Andrewes point out, every endeavor is made to disinfect this before it is allowed to pass into the sewers. The mathematical chances of detecting the typhoid bacillus in ordinary London sewage are, therefore, extremely remote. Assuming the typhoid bacillus to be intimately mixed with the ordinary sewage there would be only one typhoid bacillus in one-tenth of a cubic centimeter of sewage at the outfalls.

But the investigators only found it possible to work on one five thousandths of a cubic centimeter, and this only when 90 per cent.

---

1. Report on the Result of Investigation on the Microörganisms of Sewage. By J. Parry Laws and F. W. Andrewes. I am indebted to J. Ed. Worth, M. Inst. C. E., District Engineer, London County Council, for a copy of Messrs. Laws and Andrewes's study.

of the organisms were inhibited by the addition of 0.05 per cent. carbolic acid and incubation at 37° cent.

These considerations were so discouraging that it was determined to work on sewage from a fever hospital, and arrangements were accordingly made to allow the dejections from forty patients in the Eastern Hospital, at Homerton, to pass into the hospital sewer for two days without disinfection. A series of samples were then taken and cultivations made therefrom at once. Without going further into the detail, it may be stated that from the whole series of samples of sewage taken under these circumstances only two colonies of the bacillus of typhoid fever were certainly differentiated. In their summation Messrs. Laws and Andrewes say :

> We must be content to have shown that in the drain from the typhoid block of a fever hospital, when the stools had not been disinfected for two days, a bacillus can be found which, so far as demonstration can go, is identical with that believed to be the actual cause of typhoid fever. So far as we are aware, this important fact has never been previously demonstrated.

It is because of technical difficulties of this sort that I am not willing to admit that mere failure to find the typhoid bacillus in Lake Erie water is any special proof of its absence. At the same time I do not overlook the beneficial effects of sedimentation in the reservoirs.

Returning to the study at Lorain, it was ascertained that the sewerage system of the town was completed in 1892, and a considerable number of water-closet connections were made in October and November of that year. Previous to 1892 the deaths from typhoid fever were only such a number as may be expected in the stated population when no special contamination of the water supply exists. The few deaths actually occurring were in the fall of the year, the normal season of typhoid fever. This fact leads to the conclusion that for the small amount of sewage then contributed to the Black river by the city of Elyria, the self-purifying agencies of the river and lake were sufficient, and even with the extremely unfavorable location of the intake no serious contamination took place ; but the contribution of even a small amount of raw sewage near the water works intake was sufficient to destroy at times the small margin of safety which had previously existed.

A further interesting fact of these Lorain typhoid epidemics is that during the winters of 1892 and 1895 Lake Erie was frozen over and remained closed for several weeks, during which time we must conclude that because of the removal of the antagonistic agency of the aërobian forms of minute life, the bacillus of typhoid fever attained far greater development in Lake Erie water than is possible when the lake is open. The closing of the lake, then, by ice must be considered, the same here as at Zurich, a source of danger to any town using the raw water for domestic purposes. Taking into account that thus far the Lake Erie water supplies are all unfiltered and that in some years the lake is closed as much as three months, we are forced to the conclusion that this one consideration is a serious menace to every water supply on the lake.

As to the remedy to be applied to Lake Erie water, sand filtration is the only proper one thus far worked out. By this I mean filtration through broad beds of sand and not by mechanical filters. This method of filtration has been in use abroad for so long a time as to accumulate a body of experience which adapts it to every conceivable situation. Indeed, it has been used at the cities of Poughkeepsie and Hudson, in this state, successfully for twenty-five years.

It may be pointed out, however, that the fine clay silt, to which we have referred as present after every storm, complicates the problem of filtration of Lake Erie water to the extent that apparently settling basins of large area are a necessary adjunct of any filtration plant here.

The sands of the lake beach while clean are still not quite of the proper quality for efficient filtration as at present understood. Their use would greatly decrease the cost of a filter plant in comparison with what it would be if necessary to transport the sand from the ocean beach.

The management of the fine clay silt and the adaptation of the local sands are, then, the two problems for the Lake Erie towns to consider, and I cannot but think that more progress will be made toward a final solution by attacking the problem seriously and with due understanding of what has been done elsewhere than by assuming, as most of the Lake Erie cities have done, that in some way (thus far unexplained) Lake Erie has been excepted from the universal laws of water pollution which prevail in all other parts of the earth.

At any rate, the latter state of mind is not conducive to useful scientific results.

The time at my disposal will not permit of further discussion of these interesting as well as vastly important municipo-economic questions. From the presentation here made we may draw the following conclusions :

1. Taking into account all the conditions, the diversity of practice between Liverpool and Zurich and the cities of the United States, is so great as to be only explainable on the basis that somewhere the municipal authorities are entirely wrong. If in Liverpool and Zurich, then the people's money has been spent extravagantly and unnecessarily. If, on the other hand, the error is on this side of the water, then the municipal authorities here have assumed very serious responsibility.

2. Personally, I have no doubt the European cities are right and ours wrong, the error here to be ascribed largely to lack of definite information among water works managers as to just what all the recent biological information really means.

3. The remedy is sand filtration of every Lake Erie water supply. The use of filtration methods abroad has placed this whole matter on a basis of definite experience, which it is folly on our part to longer ignore.

4. The cost of filtration, if properly designed and the use of water properly regulated, will not exceed about 40 cents to 50 cents per capita per annum, a sum which in view of the benefits to be derived our cities can well afford to pay.

5. The root of the whole matter will be found chiefly in improved methods of administration in the American municipalities. It may be set down as self-evident that a politically governed city will be far behind in all the arts which go to make life really safe as well as enjoyable.

